# Gradient 2D/3D Perovskite Films Prepared by Hot‐Casting for Sensitive Photodetectors

**DOI:** 10.1002/advs.202000776

**Published:** 2020-05-29

**Authors:** Hok‐Leung Loi, Jiupeng Cao, Xuyun Guo, Chun‐Ki Liu, Naixiang Wang, Jiajun Song, Guanqi Tang, Ye Zhu, Feng Yan

**Affiliations:** ^1^ Department of Applied Physics The Hong Kong Polytechnic University Hung Hom Kowloon Hong Kong

**Keywords:** 2D perovskites, hot‐casting, perovskites, photodetectors, vertical heterojunctions

## Abstract

2D Ruddlesden–Popper perovskites have attracted wide attention recently because of tunable optoelectronic properties and have been used as alternatives to their 3D counterparts in various optoelectronic devices. Here, a series of (PEA)_2_(MA)*_n_*_−1_Pb*_n_*I_3_ *_n_*_+1_ perovskite thin films is designed and fabricated by a convenient hot‐casting method to obtain gradient *n* in the films, which leads to the formation of vertical heterojunctions that can enhance charge separation in the films under light illumination. Based on a single gradient perovskite film, a highly sensitive and stable photodetector with a responsivity up to 149 AW^−1^ and a specific detectivity of 2 × 10^12^ Jones is obtained. This work paves a way to realizing high‐performance optoelectronic devices with enhanced charge separation by introducing compositional gradient in a perovskite film.

## Graphical abstract

By using a unique hot‐casting method, gradient 2D/3D (PEA)_2_(MA)*_n_*_−1_Pb*_n_*I_3_ *_n_*_+1_ perovskite films with continuous changes of *n* value can be prepared, leading to vertical heterojunctions in the films. Photodetectors based on the perovskite films show high responsivity and detectivity, which can be attributed to the separation of photoexcited electron–hole pairs by the cascade band structure in the vertical heterojunctions.

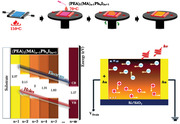

## 1 Introduction

Photodetector, which harvests light and converts it into electrical signals, is one kind of essential optoelectronic device extensively used in fields of environmental monitoring, imaging, optical communication, and biomedical sensing.^[^ ^1^, ^2^, ^3^, ^4^, ^5^, ^6^, ^7^ ^]^ The commercialized devices are predominantly made by semiconductors, including PbTe, GaN, HgCdTe, and InGaAs.^[^ ^8^, ^9^, ^10^, ^11^ ^]^ Organic–inorganic halide perovskites have been regarded as promising candidate materials for high‐performance photodetectors because of their large light absorption coefficient, long carrier diffusion length, high carrier mobility, direct bandgap, and low exciton binding energy.^[^ ^12^, ^13^, ^14^, ^15^, ^16^, ^17^, ^18^, ^19^, ^20^ ^]^ However, there are still challenges for conventional 3D perovskite materials (e.g., MAPbI_3_; MA = methylamine) to realize a satisfactory stability in ambient air.^[^ ^21^, ^22^, ^23^, ^24^ ^]^ 2D layered perovskite materials (also known as Ruddlesden–Popper phases)^[^ ^25^ ^]^ have the general formula of (RNH_3_)_2_ A*_n_*_−1_M*_n_*X_3_ *_n_*_+1_ (*n* = 1, 2, 3, 4……), where RNH_3_ is a large aliphatic or aromatic alkylammonium spacer cation, A is a monovalent organic cation, M is a divalent metal cation, X is a halide anion, and *n* represents the number of [MX_6_]^4−^ octahedral layers within each quantum well.^[^ ^26^, ^27^ ^]^ In contrast to conventional 3D perovskites, the large spacer cations of Ruddlesden–Popper perovskites (e.g., (PEA)_2_(MA)*_n_*_−1_Pb*_n_*I_3_ *_n_*_+1_; PEA = phenethylamine) enable the growth of protective layers to block moisture and oxygen from surrounding environment.^[^ ^28^, ^29^, ^30^, ^31^, ^32^ ^]^ More importantly, while 3D counterparts (e.g., MAPbI_3_) show a fixed band with conventional ambipolar characteristics,^[^ ^33^, ^34^, ^35^, ^36^, ^37^ ^]^ the 2D perovskite films consisting of alternating layers of inorganic [MX_6_]^4−^ sheets (well) and large organic spacers (barrier) offer possibilities for tuning their optoelectronic properties.^[^ ^38^, ^39^, ^40^, ^41^ ^]^

Various 2D perovskite‐based photodetectors have been reported, while the sensitivity of the devices is rather low.^[^ ^42^, ^43^, ^44^ ^]^ Due to the strong quantum confinement of Ruddlesden–Popper phases,^[^ ^45^ ^]^ the undermined performances with responsivity of 0.013 AW^−1^ through (BA)_2_(MA)*_n_*_−1_Pb*_n_*I_3_ *_n_*_+1_ (BA = butylamine) materials^[^ ^46^ ^]^ and the relatively enhanced responsivity of 0.117 AW^−1^ through (*i*BA)_2_(MA)*_n_*_−1_Pb*_n_*I_3_ *_n_*_+1_ (*i*BA = *n*‐butylamine) materials^[^ ^47^ ^]^ were reported. Meanwhile, Li et al. reported the traditional two‐step continuous deposition method dealing with the growth of BA‐based 2D superstructure perovskite on 3D perovskites, whereas the low responsivity of 0.184 AW^−1^ was achieved.^[^ ^48^ ^]^ Hwang and Lee have further developed a lateral heterostructure of 2D‐layered perovskites ((BA)_2_MAPb_2_I_7_ – (BA)_2_PbI_4_) by vapor deposition.^[^ ^49^ ^]^ Resultantly, the device exhibited the relatively high responsivity of 8.12 AW^−1^.

Here, we report the preparation of gradient 2D/3D perovskite films by a hot‐casting method, which leads to a continuously change of the 2D perovskite compositions from *n* = 1 to ∞ along the vertical bottom‐to‐top direction of the films and forms vertical heterojunctions inside them. Photodetectors based on the gradient 2D/3D perovskite films with a simple device structure were prepared, which shows a high responsivity of 149 AW^−1^, a gain of 270 and a specific detectivity of ≈2 × 10^12^ Jones. A series of perovskite thin films have been fabricated with varying predefined *n*‐values of (PEA)_2_(MA)*_n_*_−1_Pb*_n_*I_3_ *_n_*_+1_ precursor solution. At the optimum conditions, suitable 2D/3D perovskite vertical heterojunctions are created and the resultant photodetector exhibits the best performance, which could be attributed to the enhanced charge separation by 2D/3D perovskite vertical heterojunctions. The work opens a window on developing high‐performance optoelectronic devices based on 2D/3D perovskite heterojunctions by the convenient hot‐casting method.

## 2 Results and Discussion

**Figure 1:**
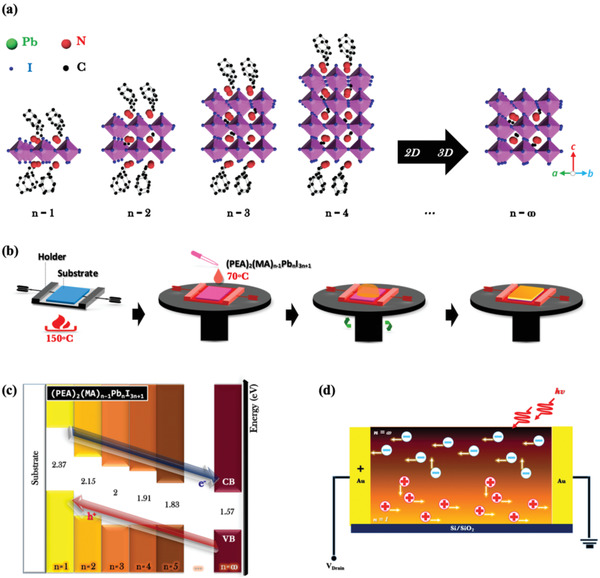
a) Schematic illustration of the crystal structure of layered perovskite materials (along [110] zone axis) with chemical formula (PEA)_2_(MA)*_n_*_−1_Pb*_n_*I_3_ *_n_*_+1_ (*n* = 1, 2, 3, 4, and ∞). b) Schematic illustration of a newly enhanced hot‐casting method presented with facile one‐step spin‐coating process for vertical 2D/3D perovskite heterojunction fabrication. c) Band energy diagram of (PEA)_2_(MA)*_n_*_−1_Pb*_n_*I_3_ *_n_*_+1_ perovskite components with different *n* numbers. d) Charge transfer diagram of a photodetector based on a gradient perovskite thin film.

The crystal structure of (PEA)_2_(MA)*_n_*_−1_Pb*_n_*I_3_ *_n_*_+1_ perovskite along [110] zone axis is shown in **Figure** **1**a, where integer *n* represents the number of [PbI_6_]^4−^ octahedral layers between organic spacer PEA^+^_._^[^ ^45^ ^]^ The material with very large *n*‐value (*n* = ∞) is plainly 3D tetragonal methylammonium lead iodide (MAPbI_3_). Figure 1b presents the solution‐based fabrication process of gradient 2D/3D perovskite films by a hot‐casting method. A substrate is fixed by an aluminum holder and heated on a hotplate. Then the holder with the substrate is coated with a perovskite precursor on a spin coater in a short time. As demonstrated in Figure S1 (see the Supporting Information), the temperature of the substrate can be maintained for a certain period of time due to the large specific heat capacity of aluminum (i.e., 0.9 kJ kg^−1^ K^−1^). The gradient distribution of perovskites can be realized immediately after the hot‐casting, which is more convenient than other techniques that may need additional post annealing or ligand exchange by spin‐coating. ^[^ ^50^ ^]^

The vertical 2D/3D perovskite heterostructure may lead to a cascade band structure in the film, as presented in Figure 1c. The valence band maximum and conduction band minimum both increase with the decrease of *n*‐value,^[^ ^51^, ^52^, ^53^, ^54^, ^55^ ^]^ which causes the charge separation with hole injection from large‐*n* to small‐*n* layers and the electron injection from small‐*n* to large‐*n*. As illustrated by the carrier transfer processes (Figure 1d) in a photodetector, holes and electrons transfer and recirculate many times in the bottom 2D and upper 3D perovskite layers, respectively, before recombining with opposite charges. Thus, long carrier lifetimes and large charge densities could be induced in the transport channel of the gradient 2D/3D perovskite film, which is favorable for the performance of photodetector.

**Figure 2:**
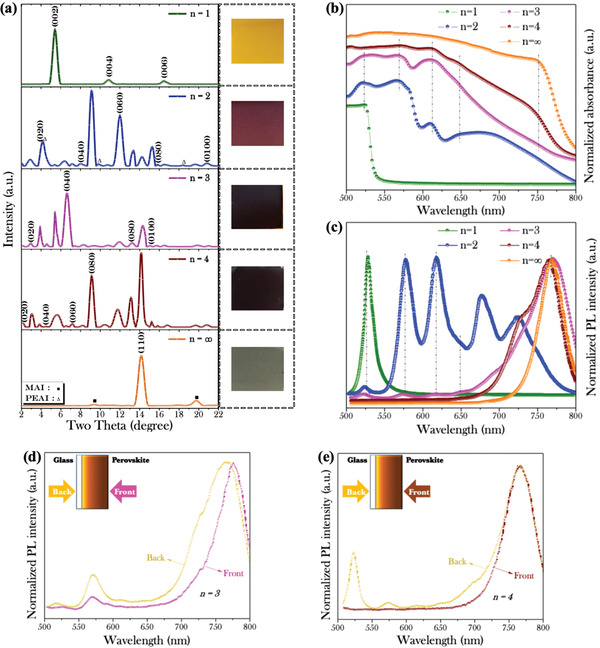
Characterization of the surface morphology of five samples by a newly enhanced hot‐casting method, which were fabricated by varying stoichiometric ratios (predefined *n*‐values = 1, 2, 3, 4, and ∞) in (PEA)_2_(MA)*_n_*_−1_Pb*_n_*I_3_ *_n_*_+1_ precursor solution. a) X‐ray diffraction (XRD) patterns. Inset: optical images of the films. b) Absorption spectra of above samples. c) Photoluminescence (PL) spectra of perovskite thin films with *n* ^+^Si/SiO_2_ substrates. Comparative PL spectra of the samples fabricated by predefined *n*‐values of d) 3 and e) 4. The perovskite thin films are illuminated from the front and back sides (as illustrated in the insets) under 488 nm laser.

To realize perovskite films with different *n*‐values, the precursor solutions consisting of phenethylammonium iodide (PEAI), methylammonium iodide (MAI), and lead iodide (PbI_2_) at a specific stoichiometric ratio of 2: *n*‐1: *n* (*n* = 1, 2, 3, 4, and ∞) in dimethyl sulfoxide (DMSO)/*N*,*N*‐Dimethylformamide (DMF) (1: 14 volume ratio) mixture are prepared. As‐grown perovskite thin films on the glass substrates present significant change in color by different predefined *n*‐values, as shown in the inset of **Figure** **2**a. When *n*‐value number is increased from 1 to 4, the color of (PEA)_2_(MA)*_n_*_−1_Pb*_n_*I_3_ *_n_*_+1_ thin films changes from yellow to black. However, when *n*‐value number is increased from 4 to ∞, the color of resulted film turned into light gray.

To evaluate the crystal structural of the perovskite films, we carried out X‐ray diffraction (XRD) measurements on the samples, as shown in Figure 2a. For *n* = 1, the perovskite film only shows (002), (004), and (006) facets of (PEA)_2_PbI_4_ perovskite phase,^[^ ^54^, ^56^, ^57^ ^]^ indicating the [PbI_6_]^4−^ sheets parallel with the glass substrates.^[^ ^51^, ^57^ ^]^ For *n* = ∞, the film only shows the (110) facet of the MAPbI_3_ perovskite and two peaks for MAI. So the perovskite film has a preferred orientation along (110) direction.^[^ ^58^ ^]^ For predefined *n*‐values of 2, 3, and 4, the films present multiphase diffraction patterns of *n* = 1 – ∞ perovskite phases, indicating rather complicated crystal structures inside them.^[^ ^38^ ^]^ Notably, some peaks for different perovskite phases are very close and cannot be differentiated clearly.^[^ ^51^, ^57^ ^]^ In addition, the diffraction peaks from both PEAI and MAI residues can be hardly observed, suggesting the high quality of the perovskite films.

The light absorption of the perovskite films are characterized and shown in Figure 2b. Similar to the XRD spectra, the absorption spectra of the perovskite films indicate that the films with predefined *n*‐values of 1 and ∞ have the single phases of PEA_2_PbI_4_ and MAPbI_3_ perovskites,^[^ ^58^, ^59^ ^]^ respectively. For the perovskite film with predefined *n*‐value of 2, four obvious absorption peaks centered at 525, 573, 610, and 675 nm corresponding to *n* = 1, 2, 3, and other quasi‐2D perovskite phases can be observed.^[^ ^58^, ^59^ ^]^ For predefined *n*‐value of 3 and 4, several absorption peaks can be observed as well.

Figure 2c shows the photoluminescence (PL) spectra of the perovskite films characterized from their top surfaces. For predefined *n*‐value of 2, six PL peaks located at 527, 576, 616, 650, 678, and 724 nm for *n* = 1, 2, 3, 4, 5, and 6 perovskite phases,^[^ ^57^, ^58^ ^]^ respectively, are obtained. When *n*‐value number is increased from 2 to 4, the PL peaks from 2D perovskite phases are weakened and a relatively strong peak from 3D perovskite phase is obtained, which is consistent with the strengthened XRD peak from (110) facet of the MAPbI_3_ perovskite. However, by using predefined *n*‐value of 4, the disappeared PL peaks from 2D perovskite phases suggest that the low‐dimensional components could majorly locate at the bottom.

To further investigate the phase distribution in the films, the PL spectrum of the perovskite film with predefined *n*‐value of 3 was characterized from the bottom surface through the glass substrate, as illustrated in the inset of Figure 2d. The PL peaks from *n* = 1, 2, 3, and other quasi‐2D perovskite phases are strengthened, while the PL peak from the 3D perovskite phase shows a slightly blueshift. In comparison, a control sample prepared by the normal thermal annealing method without the aluminum holder did not show the obvious difference in the PL spectra characterized from the top and bottom of the film (see Figure S2a, Supporting Information), indicating the phase separation is not obvious in the control sample. Similar result can be observed for predefined *n*‐values of 4 (Figure 2e). In this case, several emission peaks from *n* = 1, 2, 3, and ∞ phases are clearly observed from the back of the film, which is completely different from the spectrum measured from the front‐excitation. Meanwhile, only a single PL peak is obtained from the front‐ and back‐excitations of the control sample (see Figure S2b, Supporting Information). Therefore, for predefined *n*‐values of 3 and 4, 2D perovskite phases accumulate at the bottom layer, while 3D perovskite phase is close to the upper surface, indicating the formation of 2D/3D vertical heterojunctions in the perovskite films. Notably, the strong phase separation of the 2D/3D perovskite film cannot be achieved by the conventional thermal annealing approach. Considering the solubility of PEAI in the polar organic solvent being lower than that of MAI, the fast evaporation of organic solvent can lead to the deposition of 2D perovskites earlier than 3D counterpart. Hence, the resultant 2D/3D gradient composition can be attributed to the high temperature (≈150 °C) during the spin‐coating process, which can quickly evaporate DMSO/DMF mixture and crystallize PEA‐based 2D perovskites at the bottom surface.

**Figure 3:**
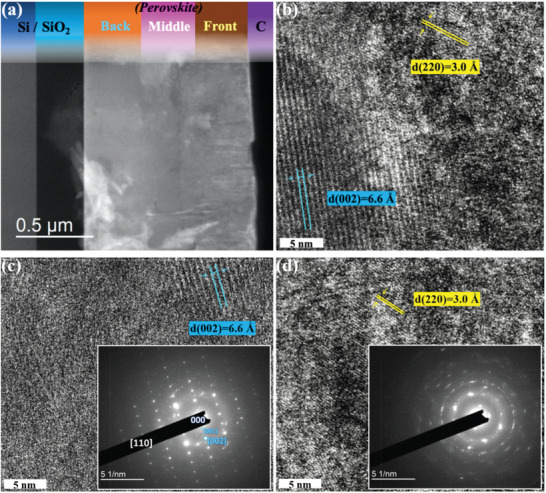
Cross‐sectional FIB‐TEM investigations of the sample fabricated by predefined *n*‐value of 3. a) The cross‐sectional FIB‐STEM image. b) The HRTEM image from middle region of the perovskite layer with the scale bar for 5 nm. The planes with lattice distances of 6.6 and 3.0 Å demonstrate the existences of 2D (blue, close to the back region) and 3D (yellow, close to the front region) perovskites, respectively. The HRTEM images from c) back and d) front regions of the perovskite layer with the scale bars for 5 nm. Two below insets show their SAED patterns, respectively.

Next, the cross‐sectional view of a perovskite film was observed under transmission electron microscope (TEM), as shown in **Figure** **3**a. We can find that 2D and 3D perovskite phases mainly locate in the back and front regions of the film, respectively. On the basis of brightness variation, the electron energy loss spectroscopy images (see Figure S3, Supporting Information) further demonstrate that more [PbI_6_]^4−^ (green for iodine)/less PEA^+^ (blue for carbon) ions locate at the front layer, suggesting that more 3D/less 2D perovskite component exists at the top.^[^ ^59^ ^]^ Notably, the contrast of C map has diminished as a result of the basal carbon film on copper TEM grids. In the middle region of the high‐resolution transmission electron microscopy (HRTEM) image presented in Figure 3b, we also can find clear phase separation of 2D and 3D perovskites. The typical periodic PEA_2_PbI_4_ (close to the back region) and MAPbI_3_ (close to the front region) perovskite phases with plane distances of 6.6 and 3.0 Å are observed in the left and right parts of the figure, respectively.^[^ ^60^ ^]^ In the back and front regions presented in Figure 3c,d, we can only find the single phase of 2D (PEA_2_PbI_4_) and 3D (MAPbI_3_) perovskites, respectively. Their selected‐area electron diffraction (SAED) patterns are also presented in the insets of the figures.^[^ ^61^, ^62^ ^]^ However, 2D perovskites with *n* ≥ 2 cannot be observed in the TEM image presumably due to the damage or disorder induced by inevitable local heating in the specimens with focused ion beam (FIB) milling.^[^ ^63^, ^64^, ^65^ ^]^

**Figure 4:**
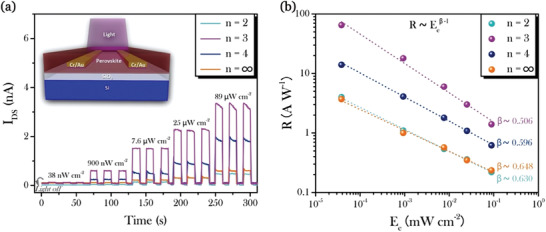
Design and performance of the devices based on varying stoichiometric ratios (predefined *n* = 2, 3, 4, and A74F) in (PEA)_2_(MA)*_n_*_−1_Pb*_n_*I_3_ *_n_*_+1_ precursor solution. a) Drain–source current versus time (*I*_DS_ *–t*) curves at varying light intensity of light with 598 nm wavelength. The inset is the schematic illustration of perovskite photodetectors. b) Responsivity versus light intensity (*R–E*_e_) curves of the devices. Drain–source voltage (*V*_DS_) is 4 V and the dotted lines in *R–E*_e_ curves are fitting curves with a formula of *R* ∝ *E*_e_^*β*−1^.

Perovskite photodetectors based on (PEA)_2_(MA)*_n_*_−1_Pb*_n_*I_3_ *_n_*_+1_ films (**Figure** **4**a, inset) were then prepared by the hot‐casting method. Cr/Au electrodes were deposited by magnetron sputtering on a n^+^Si/SiO_2_ substrate and patterned by photolithography, followed by the hot‐casting of a perovskite film. The drain current versus time (*I*_DS_–*t*) curves between the two Au electrodes of fabricated photodetectors were measured at the drain voltage (*V*_DS_) of 4 V in the dark. Then, the perovskite photodetectors were characterized under incident illumination with different intensity, as shown in Figure 4a. The device based on pure 2D PEA_2_PbI_4_ shows no response to the light illumination. For the devices based on perovskites with predefined *n*‐values from 2 to ∞, repeatable on‐off switching can be observed in three‐cycle tests for each light intensity. The photocurrent increases with increasing *n*‐value from 2 to 3, and then decreases with increasing *n*‐value from 3 to ∞. So the 2D/3D heterojunctions formed in the perovskite layer plays an important role on the device response. The response time of a photodetector is a critical issue for its practical application. Notably, the rise (*τ*_r_) and decay (*τ*_d_) time of the device are usually defined as the time taken for the current increase from 10% to 90% of steady‐state photocurrent and vice versa, accordingly. The rise/decay time of the device with predefined *n =* 3 is determined to be 69/103 ms (see Figure S4, Supporting Information).

The responsivity (*R*) of a photodetector is given by^[^ ^66^ ^]^

(1) $$R\left(AW^{-1}\right)=\frac{I_{\mathrm{Photo}}}{E_{e}WL}$$

where *I*_Photo_ is the photocurrent given by *I*_Photo_ = *I*_Light_  − *I*_Dark_. *I*_Light_ and *I*_Dark_ are the channel currents measured under light illumination and in the dark, respectively. Furthermore, *E*_e_ is the light intensity; *W* and *L* are the channel width and length of the device, respectively. Figure 4b shows the responsivity of all perovskite photodetectors as a function of illumination power with the maximum value of ≈100 AW^−1^. The responsivity of all the devices increases with the decrease of light intensity, following a relationship given by *R* ∝*E*_e_^*β*−1^ reported before.^[^ ^67^, ^68^, ^69^, ^70^ ^]^ The photocurrent and responsivity of the device with predefined *n*‐value of 3 show the highest values among the devices at the same measurement condition. For a control device prepared with the normal thermal annealing method, the responsivity is one order of magnitude lower because of the lack of vertical heterojunction formed in the perovskite film (see Figure S5, Supporting Information).

The high responsivity can be attributed to the effective spatial separation of photoexcited electron–hole pairs in the vertical heterojunction as mentioned above. To directly observe the separation of electrons and holes in perovskite films, we prepared a phototransistor with *n* = 3 perovskite layer on a n^+^Si/SiO_2_ substrate and measured the channel current under light illumination. Notably, the modulation of the channel current in a field effect transistor under a gate voltage is mainly due to the change of carrier concentration close to the gate dielectric (i.e., SiO_2_). As demonstrated in Figure S6 (see the Supporting Information), the channel current decreases with the increase of gate voltage (*V*_G_), indicating that the perovskite layer close to the substrate is p channel under light illumination. Therefore, it is reasonable to conclude that holes tend to accumulate in the bottom 2D perovskite while electrons diffuse to the top 3D perovskite with spatial charge separation.

In addition, the specific detectivity (D*) is the key parameter of a photodetector and given by^[^ ^66^, ^71^, ^72^, ^73^ ^]^

(2) $$D^{*}=\frac{{\left(AB\right)}^{1/2}}{NEP}\;$$

(3) $$NEP=\frac{i_{n}^{2^{{}^{--1/2}}}}{R}\;$$

where *A* is the effective area of the photodetector, *B* is the bandwidth, *NEP* is the noise equivalent power, $i_{n}^{2^{{}^{--1/2}}}$ is the root mean square value of the noise current and *R* is the responsivity of the device. The noise level per unit bandwidth (1 Hz) of the best performance device (predefined *n* = 3), which exhibited both the largest *I*_Photo_ and *R* among all the devices, was measured to be ≈0.2 pA Hz^−1/2^ (see Figure S7, Supporting Information). Therefore, D* of the device at the wavelengths of 598 nm is calculated to be above 2 × 10^12^ Jones (cm Hz^1/2^ W^−1^).

**Figure 5:**
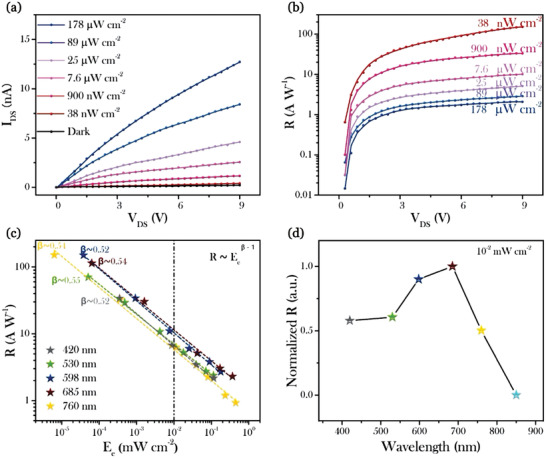
Photoresponse properties of the device based on a special stoichiometric ratio (predefined *n* = 3). Plot of a) drain–source current and b) responsivity of the device as functions of *V*_DS_ under different illumination power of light with 598 nm wavelength. Plot of responsivity as functions of c) the light intensity under different wavelengths and d) the wavelength under illumination intensity of 10^−2 ^mW cm^−2^. The dotted lines in *R–E*_e_ curves are fitting curves with a formula of *R* ∝ *E*_e_^*β*−1^.

**Figure** **5**a shows the drain current versus voltage (*I*_DS_–*V*_DS_) curves of the device with predefined *n*‐value of 3 under illumination of different intensity at the wavelength of 598 nm. Figure S8 (see the Supporting Information) exhibits the linear drain photocurrent versus voltage (*I*_Photo_–*V*_DS_) curves, demonstrating a good ohmic contact between the perovskite film and the Au electrodes. The similar results and the highest on‐off ratio of ≈100 have also been demonstrated for the wavelength of 685 nm (see Figure S9, Supporting Information). Figure 5b presents the responsivity of the device as a function of applied voltage for different light intensity. The maximum *R* of 149 AW^−1^ can be obtained at the lowest illumination intensity of 38 nW cm^−2^ under a bias of 9 V, which is higher than most of the perovskite photodetectors shown in Table S1 (see the Supporting Information). In this case, the gain *G* of the device is estimated to be 270, according to the following equation^[^ ^37^ ^]^

(4) $$G=\frac{Rhc}{e\lambda }$$

where *h* is the Planck's constant, *c* is the speed of light, *e* is the elementary charge, and *λ* is the wavelength of the incident light. The high gain can be attributed to the separation of electrons and holes by the cascade band structure in the gradient 2D/3D perovskite film, where photocarriers can circulate lots of times (equal to the gain) in the channel before recombining with opposite charges.

The device was also characterized under light with different wavelengths, including 420, 530, 685, and 760 nm. The responsivity versus voltage (*R*–*V*_DS_) curves at varying light intensity are shown in Figure S10 (see the Supporting Information). The responsivity as a function of light intensity for different wavelengths is summarized in Figure 5c, which can be fitted with the relationship *R* ∝ *E*_e_^*β*−1^ (*β* is between 0.52 and 0.55) for all wavelengths. However, the device shows a little response to the infrared light at the wavelength of 860 nm beyond the absorption edge of the perovskite film. Figure 5d shows the responsivity of the device as a function of wavelength under the same illumination intensity of 10^−2 ^mW cm^−2^. Broadband photoresponse of the device can be observed and the maximum responsivity is obtained at around 685 nm.

## 3 Conclusion

In summary, 2D/3D perovskite films with phase separation are successfully prepared by a convenient hot‐casting method. Vertical heterojunctions are formed in a single perovskite film from the bottom to the top. Highly sensitive photodetectors based on the 2D/3D perovskite films are realized for the first time. Due to the efficient charge separation in the heterojunctions, the devices show high sensitivity and fast response speed. Under optimum conditions, the device shows a high responsivity up to 10^2^ AW^−1^, a gain up to 10^2^, a specific detectivity of 2 × 10^12^ Jones, and a response time of about 0.1 s. This work paves a way of realizing a type of highly sensitive photodetector based on perovskite vertical heterojunctions in a single film.

## 4 Experimental Section

##### Materials Synthesis

Different (PEA)_2_(MA)*_n_*_−1_Pb*_n_*I_3_ *_n_*_+1_ precursor solutions were prepared by dissolving PEAI (Greatcell Solar Ltd), MAI (Greatcell Solar Ltd), and PbI_2_ (Sigma‐Aldrich) at a specific stoichiometric ratio of 2: *n* −1: *n* (*n* = 1, 2, 3, 4, and ∞) in a DMSO (Sigma‐Aldrich)/DMF (Alfa Aesar) (1:14 volume ratio) mixture, where the total Pb^2+^ molar concentration is 1.5 m. The precursor solutions were then magnetically stirred at 70 °C in the nitrogen‐filled glovebox overnight.

##### Device Fabrication

The n^+^Si/SiO_2_ (300 nm) and glass substrate was ultrasonically cleaned sequentially in deionized water, acetone, and isopropyl alcohol and dried under a stream of nitrogen gas. Cr (10 nm)/Au (100 nm) electrodes with the channel width (W) and length (L) of 800 and 4 µm, respectively, were patterned via photolithography and magnetron sputtering on the n^+^Si/SiO_2_ substrate. In the case of employing hot‐casting method during the perovskite thin film fabrication, prior to the spin‐coating, the substrate was exposed to O_2_ plasma for 5 min and preheated at 150 °C for 9 min together with an aluminum holder. After that, 60 µL of precursor solution (70 °C preheating for 30 min before use) was dropped on to the preheated substrate followed by one‐step spin‐coating process at 4000 r.p.m. for 30 s.

##### Materials Characterization

The composition and orientation of (PEA)_2_(MA)*_n_*_−1_Pb*_n_*I_3_ *_n_*_+1_ perovskite layer was confirmed by XRD (Rigaku SmartLab). The PL spectra of perovskite films were acquired by photoluminescence spectrometer (Edinburgh: FLS920) with excitation wavelength of 488 nm. The absorption spectra of perovskite films were recorded by Perkin Elmer UV–vis–NIR spectrometer. The FIB milling process and the cross‐sectional FIB‐TEM images were conducted using JEOL JIB‐4501F and JEOL JEM‐2100F TEM/STEM, respectively.

##### Electrical, Optoelectronic Measurements for the Devices

The photoresponse performance was reported using a semiconductor parameter analyzer (Keithley 4200‐SCS, Solon, Ohio, USA) under light illumination at various intensity in the nitrogen‐filled glovebox. The temporal response measurements were recorded by means of a digital oscilloscope (Tektronix TBS 2000) under a 4 V bias. The light sources were light‐emitting diodes with wavelengths of 420, 530, 598, 685, 760, and 860 nm.

## Conflict of Interest

The authors declare no conflict of interest.

## Supporting information

Supporting Information
