# Supplementary material for: Gradient 2D/3D Perovskite Films Prepared by Hot‐Casting for Sensitive Photodetectors
Source: Adv Sci (Weinh). 2020 May 29;7(14):2000776. doi: 10.1002/advs.202000776 (PMC7375231; doi:10.1002/advs.202000776)
Supplement: Supplementary file 1 — Supporting Information [file ADVS-7-2000776-s001.pdf]

Copyright WILEY-VCH Verlag GmbH & Co. KGaA, 69469 Weinheim, Germany, 2020.

Supporting Information

Gradient 2D /3D Perovskite Films Prepared by Hot-Casting for Sensitive Photodetectors

*Hok-Leung Loi, Jiupeng Cao, Xuyun Guo, Chun-Ki Liu, Naixiang Wang, Jiajun Song, Guanqi Tang, Ye Zhu, Feng Yan**

Department of Applied Physics
The Hong Kong Polytechnic University
Hung Hom, Kowloon, Hong Kong
E-mail: *apafyan@polyu.edu.hk*

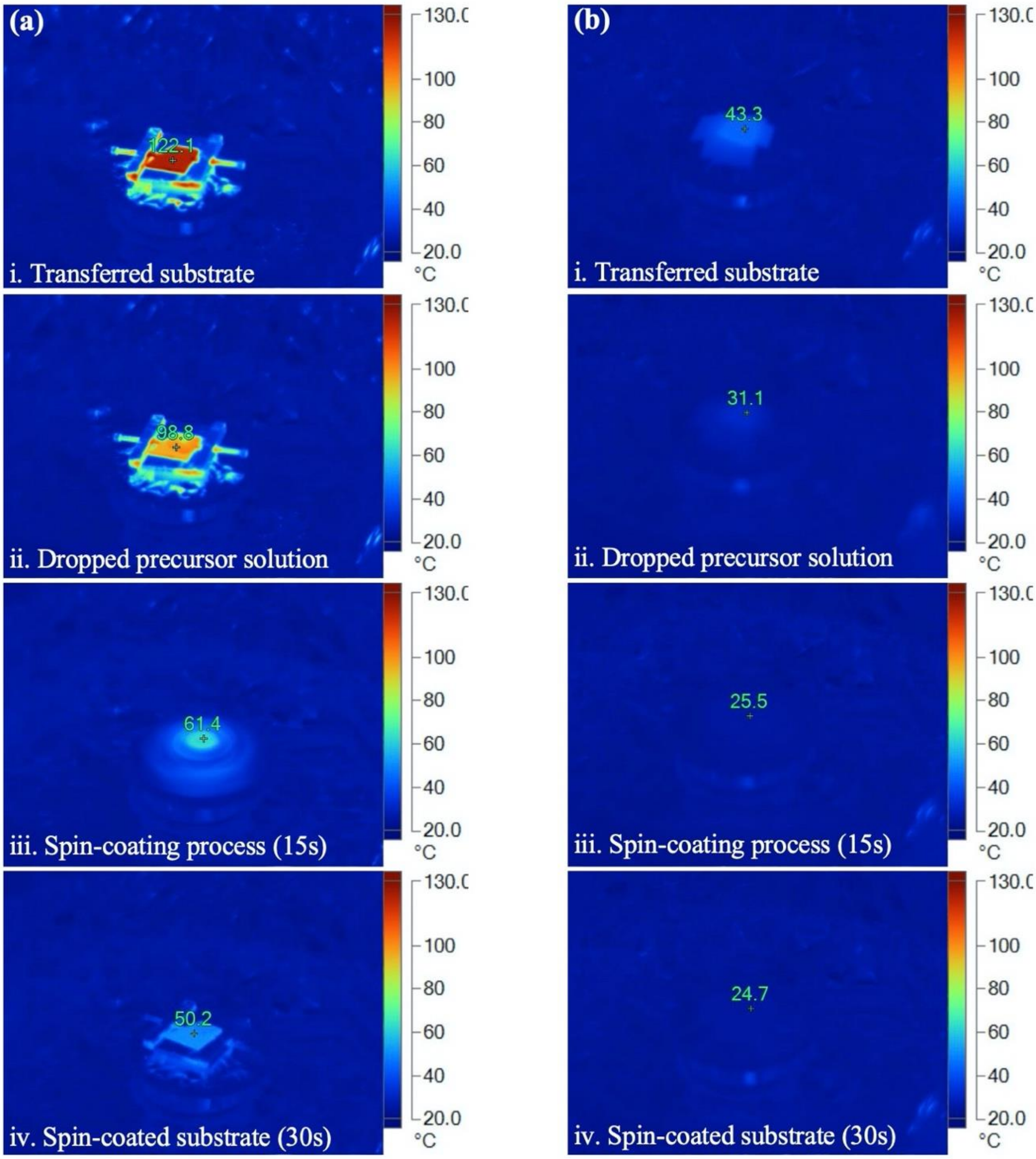

Figure S1. The infrared thermal images of **a)** modified and **b)** traditional hot-casting methods with the one-step spin-coating fabrication process for 30 s, which follows all four steps as shown in **Figure 1b**.

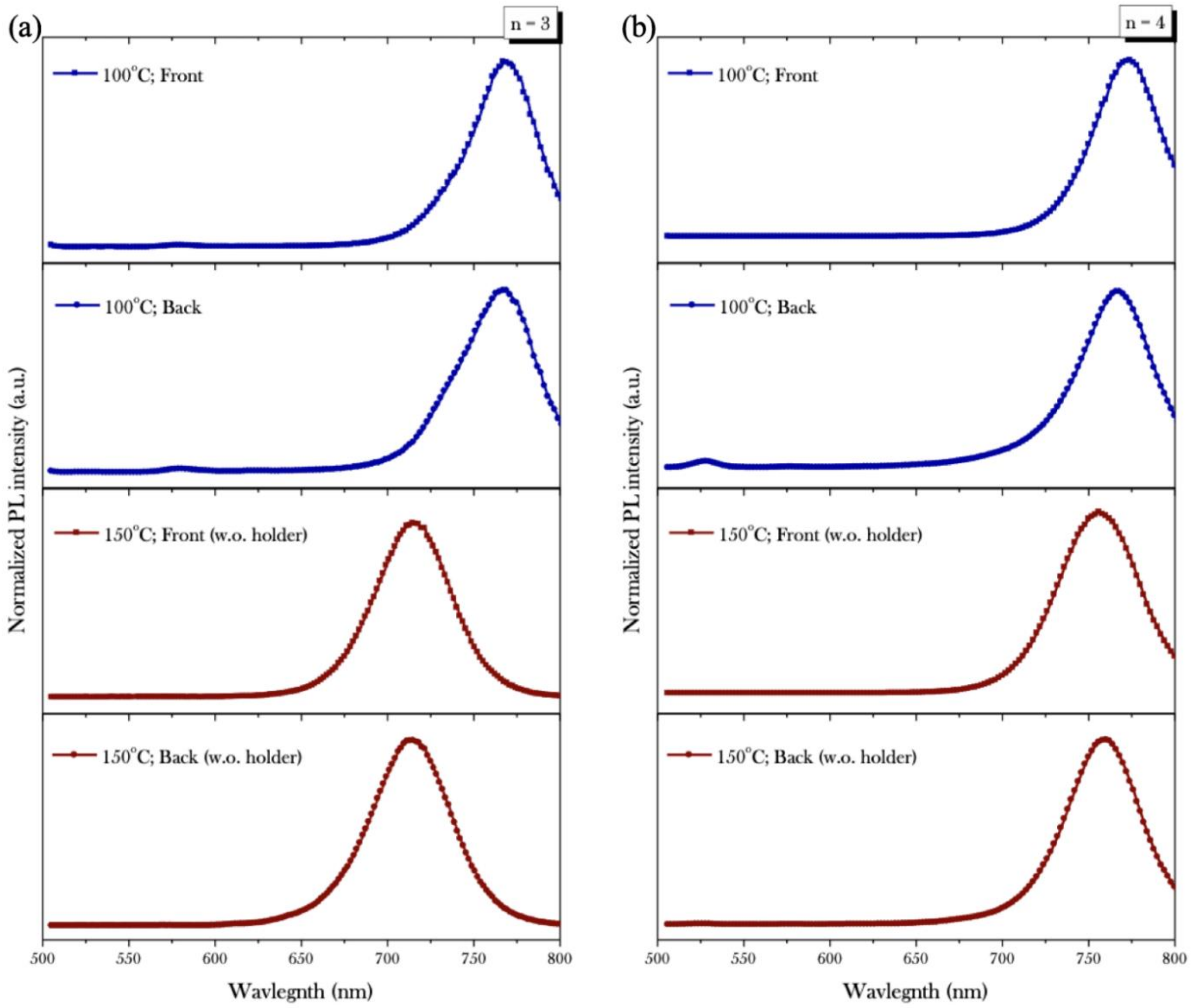

Figure S2. The comparative PL spectra of the samples fabricated by predefined *n*-values of **a)** 3 and **b)** 4 through preheat temperatures of 100 ^{o}C and 150 ^{o}C (without the aluminum holder). The elevated preheat temperature of 200 ^{o}C was found to significantly influence plasma treatment and then invalidate the following thin film crystallization. The perovskite thin films were illuminated from the front and back sides under 488 nm laser.

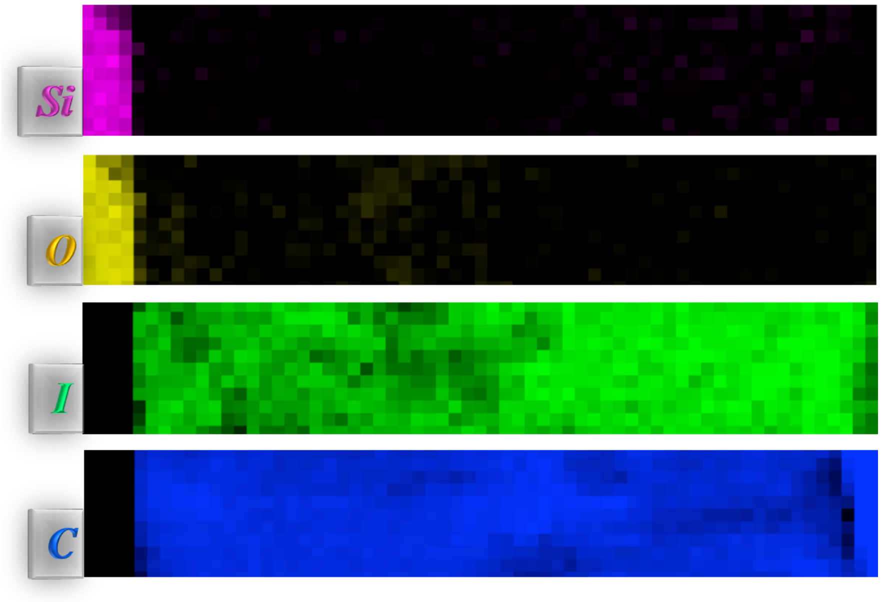

Figure S3. The Electron Energy Loss Spectroscopy (EELS) elemental maps of Si (101.1-109.5eV; purple), O (529.5-545.6eV; yellow), I (634.5-690.5eV; green), and C (281.7-302.0eV; blue) atoms from cross-sectional FIB-TEM investigations.

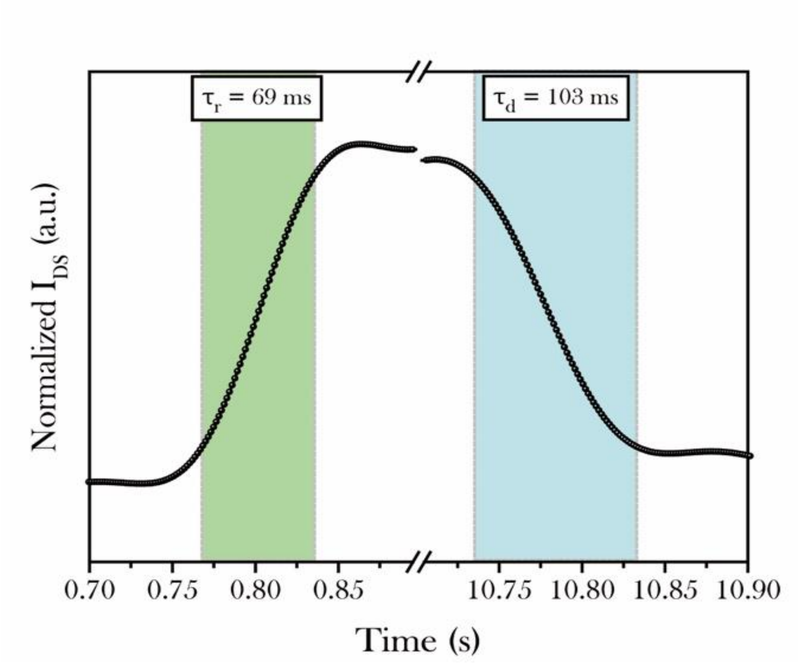

Figure S4. The temporal I_{DS} *vs.* t curve of n = 3 device in the dark and under light illumination (900 nW cm^{-2}) with 598 nm wavelength.

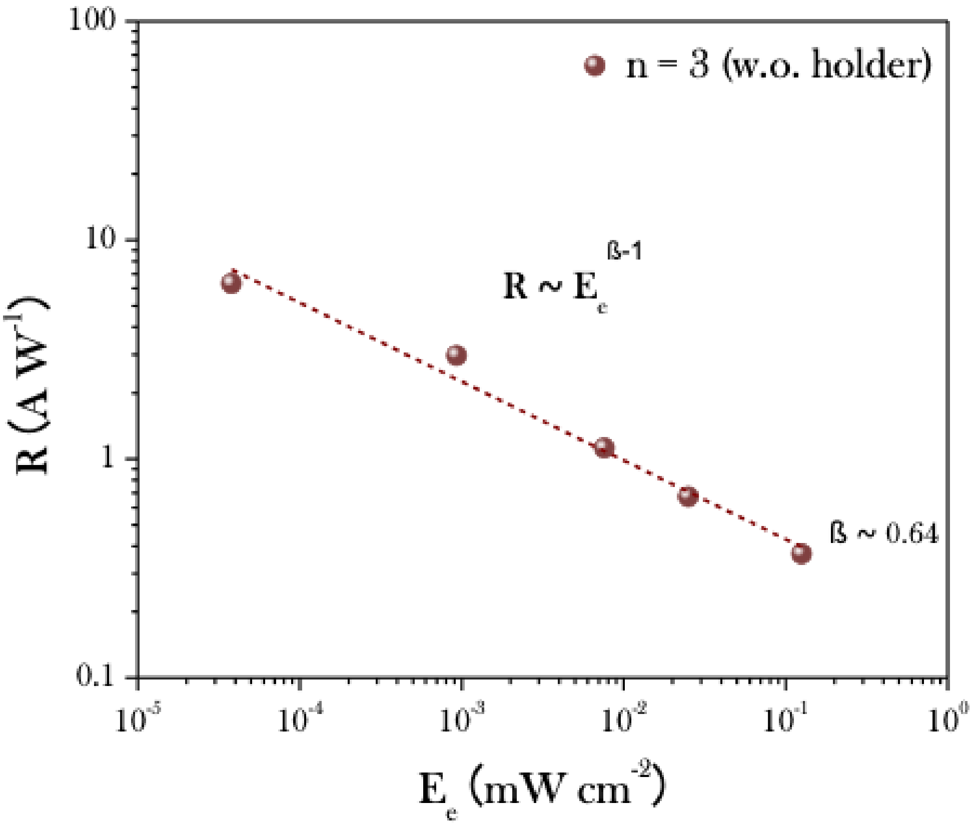

Figure S5. The R *vs.* E_e curve of control device fabricated by predefined *n*-values of 3 without preheated holder. The operating voltage is 4 V and the wavelength is 598 nm. The dotted line is fitting curve with a formula of $R \propto E_e^{\beta-1}$ under different incident illumination power.

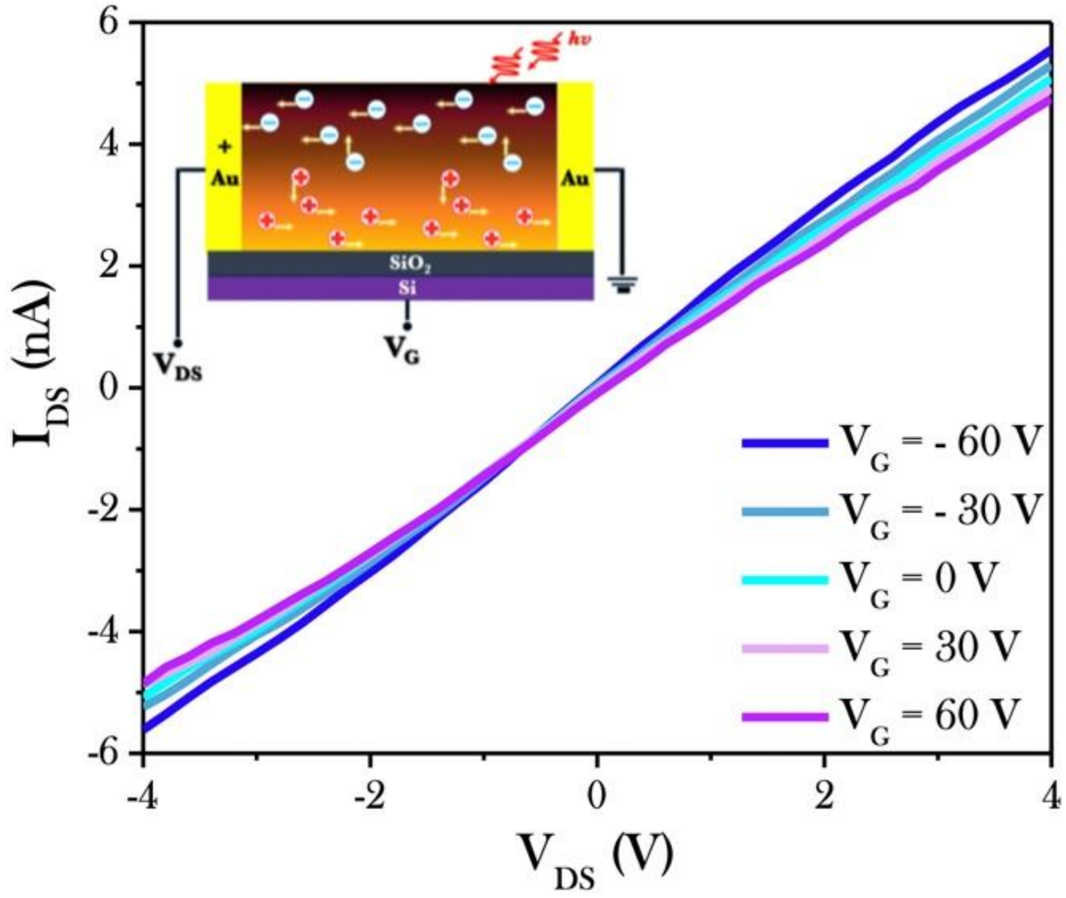

Figure S6. The I_{DS} *vs.* V_{DS} curves of n = 3 device illuminated by 598 nm light (89 μW cm^{-2}) measured under different gate voltage (V_G). Inset: device structure of the phototransistor.

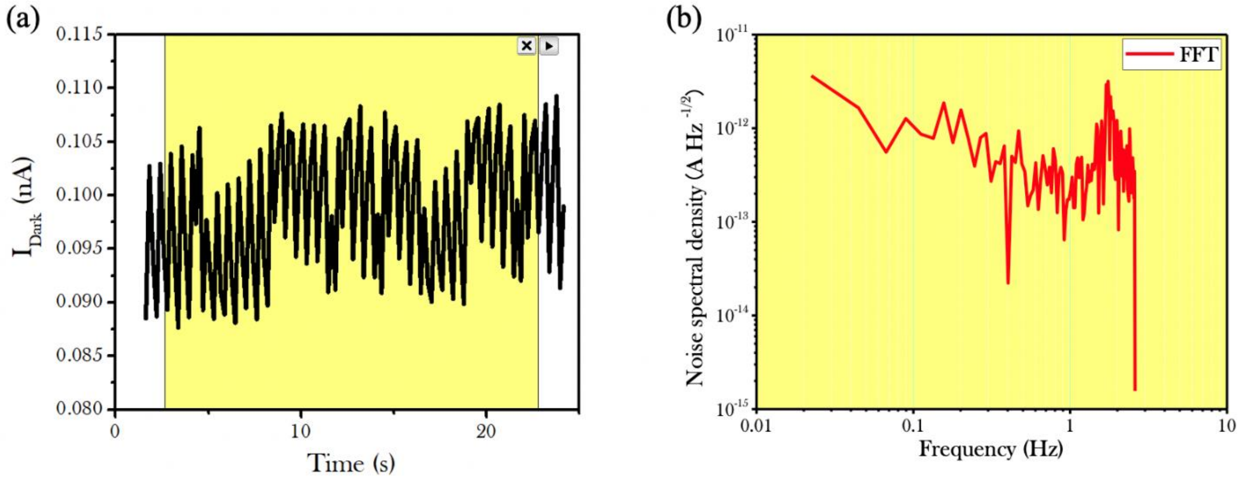

Figure S7. a) The noise of the dark current of n = 3 device. The operating voltage is 4 V. **b)** Analysis of noise spectral density of this 2D/3D perovskite photodetector by the Fast Fourier Transform (FFT) from corresponding dark current noise.

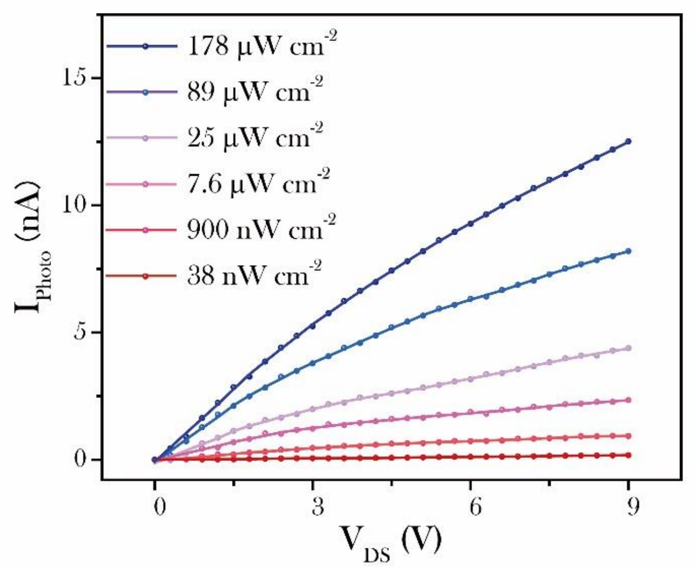

Figure S8. The I_{Photo} *vs.* V_{DS} curves of n = 3 device under varying illumination power of light at 598 nm wavelength.

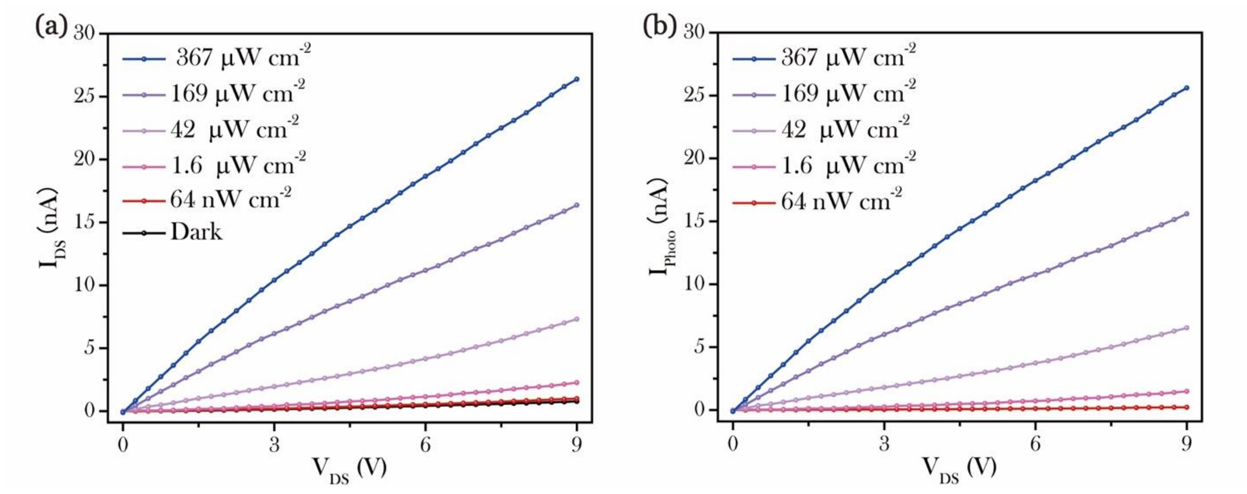

Figure S9. The **a)** I_{DS} *vs.* V_{DS} and **b)** I_{Photo} *vs.* V_{DS} curves of n = 3 device under varying illumination power of light at 685 nm wavelength.

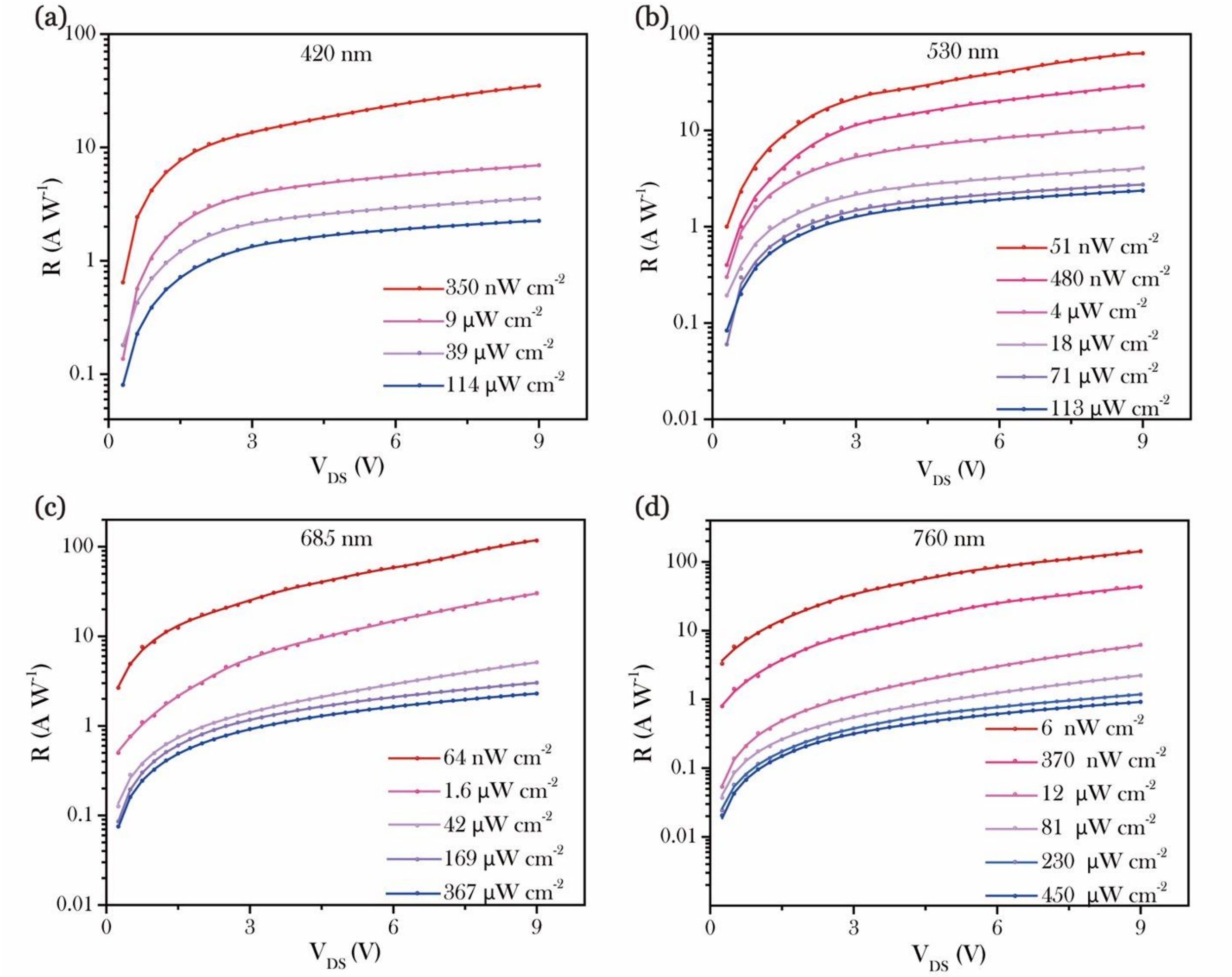

Figure S10. The R *vs.* V_{DS} curves of n = 3 device under **a)** ultraviolet (420 nm), **b,c)** visible (530 and 685 nm, respectively), and **d)** near infrared (760 nm) light illumination.

Table S1. Detailed comparison of performance parameters for 2D perovskite -based photodetectors.

Active layer	Configuration	R [A/W]	D^* [Jones]	τ_r/τ_d [ms]	On-off ratio	Wavelength [nm]	Voltage [V]	Reference
$(BA)_2MA_{n-1}Pb_nI_{3n+1}$ /$MAPbI_3$	Vertical	0.184	8×10^{10}	–	1×10^2	White light	–	[48]
$(PEA)_2MA_3Pb_4I_{13}$	Vertical	0.46	6×10^{11}	5.8/4.6	2×10^4	600	–	[41]
$(BA)_2MA_2Pb_3I_{10}$	Lateral	0.013	–	10/7.5	1×10^3	White light	30	[46]
$(iBA)_2MA_3Pb_4I_{13}$	Lateral	0.117	–	16/15	4×10^2	532	1.5	[47]
$(BA)_2MA_{n-1}Pb_nBr_{3n+1}$	Lateral	0.19	–	210/240	2×10^3	500	1	[40]
$(BA)_2MAPb_2I_7$ /$(BA)_2PbI_4$	Lateral	8.12	1.5×10^{12}	–	4×10^2	460	30	[49]
$(OA)_2FA_{n-1}Pb_nBr_{3n+1}$	Lateral	32	–	0.25/1.45	–	442	9	[42]
$(PEA)_2MA_{n-1}Pb_nI_{3n+1}$ single crystal	Lateral	0.25	8.6×10^{12}	–	–	500	5	[57]
$(PEA)_2SnI_4$	Lateral	16	1.9×10^{11}	630/3600	–	470	5	[31]
$(PEA)_2PbI_4$ single crystal	Lateral	98	1.6×10^{15}	0.06/0.05	–	460	4	[32]
$(PEA)_2MA_{n-1}Pb_nI_{3n+1}$ /$MAPbI_3$	Lateral	**149**	2×10^{12}	69/103	1×10^2	598	9	**This work**
